# Lower neutrophil‐to‐lymphocyte ratio and positive programmed cell death ligand‐1 expression are favorable prognostic markers in patients treated with pembrolizumab for urothelial carcinoma

**DOI:** 10.1002/cam4.4779

**Published:** 2022-06-14

**Authors:** Yu Miyama, Go Kaneko, Koshiro Nishimoto, Masanori Yasuda

**Affiliations:** ^1^ Department of Pathology Saitama Medical University International Medical Center Saitama Japan; ^2^ Department of Uro‐Oncology Saitama Medical University International Medical Center Saitama Japan

**Keywords:** neutrophil‐to‐lymphocyte ratio, pembrolizumab, programmed cell death ligand‐1, urothelial carcinoma

## Abstract

**Background:**

Immune checkpoint inhibitors (ICIs) are effective in some cancer patients; however, they may show no efficacy in others. Predictive biomarkers are crucial for appropriately selecting the patients who receive ICI therapy. This study aimed to clarify the predictors of disease progression in urothelial carcinoma (UC) patients treated with an ICI, pembrolizumab.

**Methods:**

We analyzed the response patterns of 50 UC patients who were treated with pembrolizumab, as well as the association between survival and clinicopathological factors. Clinical factors included age, sex, body mass index, clinical courses, laboratory data, metastases, and adverse events. Pathological factors included special variant, squamous differentiation, programmed cell death ligand‐1 (PD‐L1) expression, CD8‐positive lymphocytes density, and *CDKN2A/p16* homozygous deletion.

**Results:**

During pembrolizumab treatment, four (8%), 11 (22%), and eight (16%) patients achieved the best‐case scenarios of complete response, partial response, and stable disease, respectively. Twenty‐seven patients (54%) showed progressive disease. In this study, younger age, lower preoperative neutrophil‐to‐lymphocyte ratio (NLR), and positive PD‐L1 expression were significantly correlated with longer progression‐free survival and overall survival. Moreover, lower NLR and positive PD‐L1 expression were independently associated with longer OS in multivariate analysis.

**Conclusions:**

Based on our observations, lower NLR and positive PD‐L1 expression may be independent favorable prognostic markers in UC patients treated with pembrolizumab. These results suggest that both host and tumor status can reflect the effectiveness of pembrolizumab among patients with UC.

## Graphical abstract

Tumor status of positive PD‐L1 expression, and host immune status of low neutrophil‐to‐lymphocyte ratio, are both prognostic markers for urothelial carcinoma patients treated with pembrolizumab.

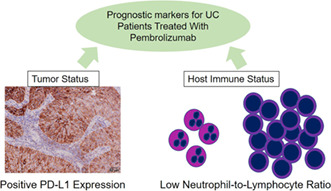

## 1 INTRODUCTION

Immune checkpoint inhibitors (ICIs) are increasingly being used in patients with advanced‐stage urothelial carcinoma (UC). However, only 20% of UC patients respond to ICIs.^1^, ^2^, ^3^, ^4^, ^5^ The selection of patients who will benefit from ICI therapy is both socially and economically important.

Several ICIs recently demonstrated efficacy and survival benefit, and they are widely used in clinical practice. Among them, pembrolizumab clearly revealed overall survival benefits over chemotherapy in the phase III study with a large sample size,^1^, ^6^ and its use is strongly recommended in the National Comprehensive Cancer Network^7^ and European Association of Urology guidelines.^8^ If the use of pembrolizumab is not possible, the use of other ICIs, such as nivolumab, avelumab, and atezolizumab, can be used.^7^, ^8^ Furthermore, erdafitinib, which is a pan‐fibroblast growth factor receptor inhibitor, is also approved by Food and Drug Administration (FDA). However, pembrolizumab is only used for patients with platinum‐resistant advanced UC who desire further treatment in the Japanese insurance system as of 2022. Therefore, we searched some prognostic markers for the UC patients treated with pembrolizumab in this study.

Recently, a number of second and later line studies on therapy reported that response rates for anti‐programmed cell death‐1 (PD‐1)/PD ligand‐1 (PD‐L1) antibodies were higher in patients with PD‐L1–high UC than in those with PD‐L1–low UC.^2^, ^9^, ^10^, ^11^ In addition, pembrolizumab, a PD‐1 antibody, was approved by the FDA for patients of solid tumor with high‐tumor mutation burden, including UC. Wang et al. developed a unique scoring classifier, tumor mutational burden‐related LASSO score (TLS) using the LASSO algorithm, which predicts response to atezolizumab, an anti‐PD‐L1 antibody. In this study, Tumor mutation burden (TMB) was significantly correlated with neoantigen, and these factors predict atezolizumab. In addition, TLS was associated with an immune‐inflamed phenotype.^12^ Similarly, CD8‐positive lymphocytic infiltration is associated with the response to ICIs.^9^ From these results, we hypothesized that such an immune inflamed tumor microenvironment contributes to the response to ICIs.

Genetic alterations frequently detected in UC could also be a prognostic marker for ICIs. Nassar et al. reported clinical and seven genomic factors correlated with clinical outcomes in univariable analysis in the ICI cohort, such as neutrophil‐to‐lymphocyte ratio (NLR), visceral metastasis, and single‐nucleotide variant (SNV) count. Especially, in genetic alterations, homologous deletion of *CDKN2A/p16* had tended to have no clinical benefit for ICIs.^13^

Some studies have further suggested associations between special variants and responses to ICIs.^14^, ^15^, ^16^ Regarding this, molecular subtypes, which reflect morphological variants to some extent, are provocative in muscle‐invasive urinary bladder. Although the effectiveness of adjuvant chemotherapy is associated with basal subtypes,^17^, ^18^ the relationship between response to ICIs and molecular subtypes is controversial. For example, in IMvigor‐210, cluster II in TCGA subtypes, which are similar to p53‐like/luminal infiltrated subtype, were associated with response to atezolizumab, whereas cluster I (luminal papillary subtype) was associated with resistance.^9^ In contrast, data from the Checkmate 275 clinical trial of nivolumab demonstrated that responses were observed in basal molecular subtype,^5^ but the association between the luminal papillary subtype and resistance was also observed.^19^ Therefore, the association between molecular subtype and response to ICIs needs to be investigated. In this context, identifying morphological variants are also important to bridge molecular subtype and response to ICIs.

This study explored predictive markers based on clinicopathological data analysis of patients with platinum‐resistant locally advanced or metastatic UC who received pembrolizumab and PD‐1 antibody therapy, pembrolizumab. First, we performed a clinical and histological review. Previously, we reported that squamous differentiation was correlated with tumor progression in patients administered with pembrolizumab for UC.^14^ In our previous study, we considered squamous differentiation and detected both morphological characteristics, such as intracellular bridges and keratinization, and MAC387 expression in immunohistochemistry. MAC387, a highly sensitive and specific marker of squamous differentiation,^20^ is especially useful when keratinization is unclear or the tumor size is small and indeterminate for squamous differentiation. Second, markers that have been reported as potential prognostic markers for response to ICI treatment were analyzed. These include an immunohistochemical analysis of PD‐L1 expression by combined tumor and immune cell scoring algorithms and an evaluation of the density of CD8‐positive lymphocytic infiltration. Third, we performed a fluorescence in situ hybridization (FISH) analysis of *CDKN2A/p16* homozygous deletion. In addition, accumulating evidence suggests that the prognostic value of tumor biomarkers may vary according to the patient's characteristics through host–tumor interactions. For example, the association of tumor PD‐L1 expression with outcomes varied among patients with low or high preoperative serum platelet counts in upper tract UC (UTUC).^21^ Then, we examined the interactive effect of tumor and host factors on the prognosis of these patients.

## 2 MATERIALS AND METHODS

### 2.1 Patients, samples, and clinical data

From February 27, 2018, to March 16, 2021, we analyzed 50 (27 with bladder urothelial carcinoma [BLCA] and 23 with UTUC) of 65 patients with platinum‐resistant locally advanced or metastatic UC who received pembrolizumab in our institution based on availability. All samples of these 50 patients enrolled in this study were acquired via surgical resection or biopsy before the patients received pembrolizumab. When a single case had multiple samples, we chose one sample, the most recent and also a surgical sample rather than a biopsy sample. We extracted the clinical data analyzed in this study from the patients' medical records. Complete blood count was assessed within 7 days of pembrolizumab treatment.

This study was approved by the Institutional Review Board of the Saitama Medical University International Medical Center (approval numbers: 20‐129 and 20‐149), and informed consent was acquired from all the patients.

### 2.2 Statistics

We analyzed the prognostic factors of progression‐free survival (PFS) and overall survival (OS) using the Cox‐proportional hazards model. PFS and OS were analyzed using the Kaplan–Meier method and a log‐rank test. These analyses were performed using Python software (Python Software Foundation, Wilmington, DW). The interaction was assessed using the least‐squares method for the cross‐product of age, NLR, PD‐L1 expression, and squamous differentiation in PFS or OS. The interaction was analyzed using JMP Pro 16.1 software (SAS Institute, Cary, NC).

### 2.3 Histological review

Histological review using hematoxylin and eosin‐stained slides was performed by a single genitourinary pathologist (YM) who was blinded to the patients' outcomes. The morphological characteristics analyzed in this study were histological grade and subtype of UC, that is, divergent differentiation shown by squamous differentiation, or types of variant morphologies (i.e., micropapillary or plasmacytoid), according to the 2016 World Health Organization classification system.^22^ Squamous differentiation was demonstrated morphologically by the presence of intercellular bridges or keratinization. In addition, we combined MAC387 expression in immunohistochemistry when the morphology showed indeterminate for squamous differentiation, as previously described.^14^

### 2.4 Immunohistochemistry

Formalin‐fixed paraffin‐embedded tumor samples were sliced into 4 μm sections for the immunohistochemical analyses. The immunohistochemistry of each marker was performed using the antibodies: MAC387 (mouse monoclonal antibody; clone MAC387; 1:4000; GeneTex, Alton, CA), PD‐L1 (rabbit monoclonal antibody; clone SP263; prediluted; Roche Diagnostics, Tucson, AZ), and CD8 (mouse monoclonal antibody; clone C8/144B; 1:40; Agilent). Antigen retrieval was performed using Cell Conditioning Solution (CC1‐buffer) (Roche Diagnostics). Visualization was performed using the OptiView DAB Universal Kit for PD‐L1 (Roche Diagnostics) and the iView DAB Universal Kit for the other antibodies (Roche Diagnostics). Hematoxylin was used for counterstaining. Immunoreactivity was assessed by the same pathologist (YM) who performed the histological review.

### 2.5 Programmed cell death ligand‐1 expression

**FIGURE 1:**
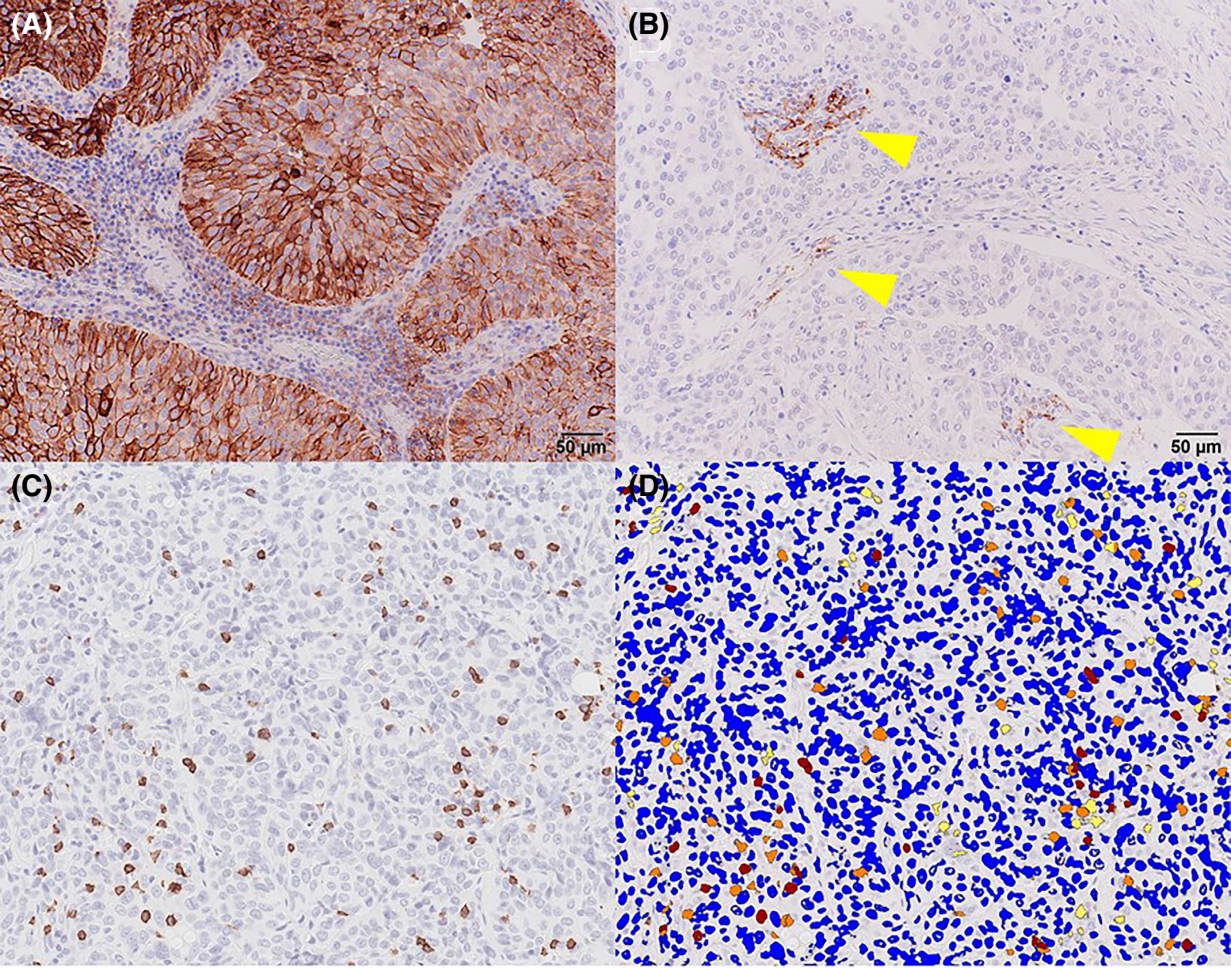
(A) PD‐L1 expression was regarded as positive. (B) PD‐L1 expression was considered to be negative. Only immune cells outside the tumor area expressed PD‐L1 (arrowheads). (C) Pre‐analytical image of CD8 immunohistochemistry. (D) Post‐analytical image shown in Figure 1C. Blue, yellow, orange, and red pixels indicate negative, weak‐positive, positive, and strong‐positive pixels of CD8 immunohistochemistry, respectively

We used the SP263 assay for PD‐L1 expression, which shows PD‐L1 staining that is highly concordant with the 22C3 assay.^23^ Based on the SP263 assay scoring algorithm for UC,^24^ we assessed the proportions of tumor cells and immune cells with staining at any intensity above a threshold; tumors were classified as PD‐L1–high if staining was ≥25% in either compartment. In cases where immune cells were 1% or less of the tumor area, tumors were classified as PD‐L1–high only if 100% of the immune cells were expressed for PD‐L1. Figure 1A,B show examples of PD‐L1 expression by immunohistochemistry.

### 2.6 CD8‐positive cells density

Image analysis of CD8‐positive cells was performed using Aperio ImageScope software (Leica Biosystems, Deer Park, IL), as previously described.^25^ Briefly, we used the Aperio nuclear algorithm, which measures the area and intensity of nuclei. Blue, yellow, orange, and red pixels indicate negative, weak‐positive, positive, and strong‐positive pixels of CD8 immunohistochemistry, respectively. The total positivity area divided by the region of interest area was regarded as the density of CD8‐positive lymphocytes. Figure 1C,D show an example of the image analysis.

### 2.7 *CDKN2A/p16* homozygous deletion

**FIGURE 2:**
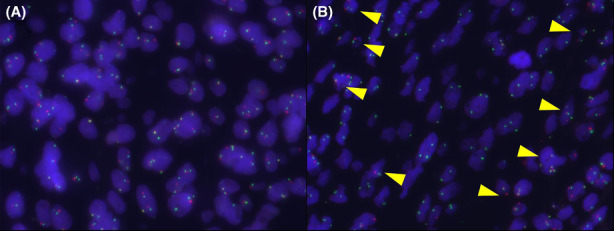
(A) Normal signal pattern. Centromere (green) and *CDKN2A/p16* (red) signals are equally observed. (B) Homozygous deletion of *CDKN2A/p16*. *CDKN2A/p16* signals (red) are not observed in tumor cells but in stromal cells (arrowheads)

To assess CDKN2A/p16 homozygous deletion in FISH, formalin‐fixed paraffin‐embedded tumor samples were used. Paraffin sections (3 μm) were baked for an hour, placed in xylene for 5 min, and dehydrated in 100% ethanol. The slides were treated with heat (95°C) for an hour with a pretreatment reagent (VP2000) before hybridization. After cooling, deproteinization was performed with 0.8% pepsin lysis buffer and 2X saline sodium citrate (SSC) buffer. Following this, dehydration was performed in 70%, 85%, and 100% alcohol solutions for 5 min each, followed by drying. Next, the ZytoDot 2 SEPC CDKN2A/CEN9 probe (ZytoVision, Fischkai, BRV) was used. DNA denaturation was performed at 80°C for 5 min and placed overnight in a hybridization chamber at 37°C. The post‐hybridization washes were performed with 2X SSC buffer/0.3% NP‐40. The slides were counterstained with DAPI stain. A minimum of 25 cells were analyzed for copy number changes in chromosome 9p21 (*CDKN2A/p16*). When ≥15 cells showed zero *CDKN2A/p16* signals, it was regarded as homozygous deletion.^26^ If no abnormalities were detected, the remaining cells were counted until 100 cells were evaluated. Figure 2 shows an example of the *CDKN2A/p16* signals of tumor cells in FISH.

## 3 RESULTS

All clinicopathological and survival data are shown in Table S1.

### 3.1 Survival and response to pembrolizumab

In this study, as of March 3, 2022, the median values of PFS and OS were 108.5 days (3.62 months) and 329 days (10.97 months), respectively. Four (8%), 11 (22%), and eight (16%) patients achieved the best‐case scenarios of complete response (CR), partial response (PR), and stable disease (SD), respectively, according to the patients' computed tomography (CT) findings (37 cases were assessed) and the Response Evaluation Criteria in Solid Tumors version 1.1 (RECIST).^27^ However, over half of the patients (n = 27, 54%) had progressive disease (PD) after pembrolizumab administration. Overall, patients who achieved CR had an extremely good prognosis, whereas the others did not.

### 3.2 Relation between survival and clinicopathological data

**FIGURE 3:**
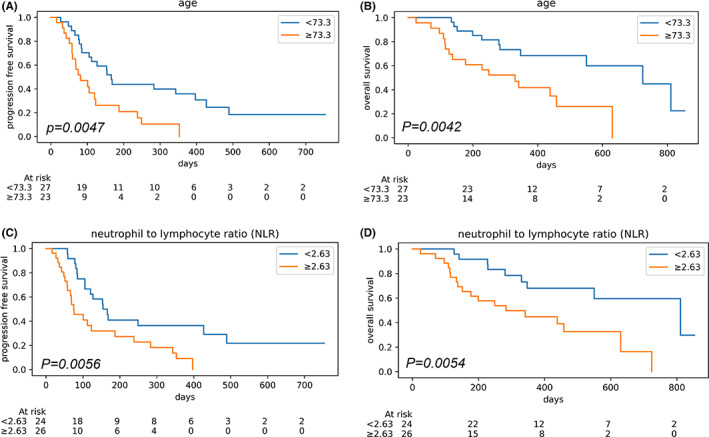
(A) Kaplan–Meier curves for progressive free survival in two groups with younger and older age. (B) Kaplan–Meier curves for overall survival in two groups with younger and older age. (C) Kaplan–Meier curves for progressive free survival in two groups with low and high neutrophil‐to‐lymphocyte ratios. (D) Kaplan–Meier curves for overall survival in two groups with low and high neutrophil‐to‐lymphocyte ratios

Subsequently, we assessed the relationship between survival and clinicopathological data (Table 1). The median age of the cohort was 71.6 (interquartile range: 69.5–77.1) years, and patients younger than 73.5 years had significantly better PFS and OS than older patients (log‐rank *p* = 0.0047 and 0.0042, respectively; Figure 3A,B). Among laboratory data, patients with a lower NLR (<2.6) had significantly better PFS and OS (log‐rank *p* = 0.0056 and 0.0054, respectively, Figure 3C,D). Metastases to the liver or presence of visceral metastases, and the presence of adverse events were correlated with poorer survival. However, these factors were associated with either PFS or OS, respectively (Figure S1).

**TABLE 1:** Patient characteristics and statistics

			Progressive‐free survival (PFS)				Overall survival (OS)					
			Logrank p	Cox p	HR	(95%CI)	Fig.	Logrank p	Cox p	HR	(95%CI)	Fig.
Response	CR vs. PR vs. SD vs. PD	(4 vs. 11 vs. 8 vs. 27)	n.a.					n.a.				
Physical												
Age	71.6 (69.5–77.1)											
	<73.3 vs. > =73.3	(23 vs. 27)	**0.0047**	*0.09*	1.69	(0.91–3.72)	**3A**	**0.0042**	*0.12*	1.57	(0.85–4.68)	**3B**
Sex	Male vs. female	(31 vs. 19 )	*0.7*					*0.95*				
Body mass index (BMI)	22.3 (19.5–23.9)											
	<22.3 vs. > =22.3	(25 vs. 25)	*0.42*					*0.61*				
Clinical courses												
Duration till pembrolizumab after recurrence	139.5 (7–802)											
	<139.5 vs. > =139.5	(25 vs. 25)	*0.73*					*0.71*				
Laboratory data												
Neutrophil‐to‐lymphocyte ratio (NLR)	2.7 (2.2–4.7)											
	<2.63 vs. > =2.63	(24 vs. 26)	**0.0056**	*0.06*	1.87	(0.97–3.88)	**3C**	**0.0054**	**0.03**	2.22	(1.12–6.75)	**3D**
Platelet‐to‐lymphocyte ratio (PLR)	189.9 (127.2–257.8)											
	<189.9 vs. > =189.9	(25 vs. 25)	*0.74*					*0.066*				
Lymphocyte‐to‐monocyte ratio (LMR)	4.1 (2.6–6.5)											
	<4.1 vs. > =4.1	(25 vs. 25)	*0.44*					*0.099*				
Metastases before pembrolizumab												
Lung metastasis	No vs. yes	(35 vs. 15)	*0.22*					*0.64*				
Liver metastasis	No vs. yes	(40 vs. 10)	*0.11*				**S1A**	**0.041**				**S1B**
Bone metastasis	No vs. yes	(44 vs. 6)	*0.062*					*0.43*				
Lymph node metastasis	No vs. yes	(25 vs. 25)	*0.96*					*0.59*				
Peritoneal metastasis	No vs. yes	(44 vs. 6)	*0.96*					*0.96*				
Visceral metastasis	No vs. yes	(23 vs. 27)	**0.0075**				**S1C**	*0.13*				**S1D**
Adverse effect (AE)	No AE vs. AE of all grade	(23 vs. 27)	**0.0013**				**S1E**	*0.098*				**S1F**
	No AE and AE of grade 1–2 vs. AE of grade 3	(42 vs. 8)	*0.35*					*0.23*				
Pathological factors												
Special variant	No vs. yes	(34 vs. 16)	*0.69*					***0.092***				
Squamous differentiation	No vs. yes	(37 vs. 13)	*0.51*					*0.17*				
PD‐L1 expression	Low vs. high	(37 vs. 13)	**0.021**	*0.06*	1.85	(0.95–4.71)	**4A**	**0.0013**	**0.01**	2.72	(1.78–35.03)	**4B**
CD8+ lymphocytes	46.2 (25.8–166.9)											
	<46.2 vs. > =46.2	(26 vs. 24)	*0.054*					*0.27*				
*p16*(*CDKN2A*) homozygous deletion in FISH	Loss vs. retained	(30 vs. 30)	*0.30*					*0.35*				

Italic indicates no significance of *p* value; Bold indicates significance of *p* value.

Abbreviations: CR, complete response; PR, partial response; SD, stable disease; PD, progressive disease; PD‐L1, programmed cell death ligand‐1.

**FIGURE 4:**
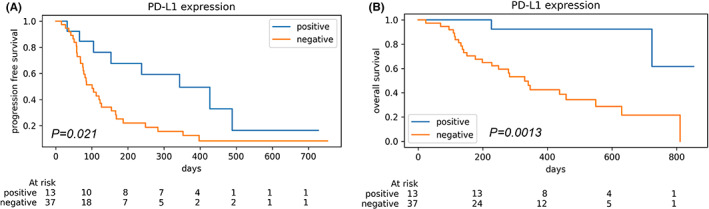
(A) Kaplan–Meier curves for progressive free survival in two groups with negative and positive PD‐L1 expression. (B) Kaplan–Meier curves for overall survival in two groups with negative and positive PD‐L1 expression

In terms of pathological data, PD‐L1 expression was observed in 13 out of 50 cases (26%) and was associated with better PFS and OS (log‐rank *p* = 0.021 and 0.0013, respectively, Figure 4A,B). The other pathological data, including special variant, squamous differentiation, CD8‐positive lymphocytes, and *p16* homologous deletion in FISH, were not associated with prognosis.

In addition, we combined these potential prognostic factors and analyzed the association with patient outcome, using the least‐squares method for the cross‐product of age, NLR, and PD‐L1 expression in PFS or OS. However, there was no interactive effect between age, NLR, and PD‐L1 expression in PFS or OS (data not shown).

## 4 DISCUSSION

In this study, younger age, lower NLR, and positive PD‐L1 expression could be favorable prognostic markers in terms of both PFS and OS. In addition, lower NLR and positive PD‐L1 expression were independently associated with longer OS in the Cox‐proportional hazards model analysis.

Several studies have reported that higher NLR is associated with poorer prognosis in patients who received pembrolizumab for UC;^28^, ^29^ this is consistent with our results. In a meta‐analysis, a higher NLR resulted in worse OS and PFS in patients administered with ICIs for solid cancers, including UC.^30^ Although there is no uniform cutoff value and the markers remain dynamic, NLR could reflect the systemic inflammation regulated by tumor and host‐derived factors.^31^

We assessed PD‐L1 expression of tumor and immune cells using the SP263 assay scoring algorithm. Positive PD‐L1 expression was associated with better OS and PFS. Consistent with our results, PD‐L1 expression appears to be prognostic in the context of second‐line therapy.^3^, ^10^ In addition, Powles et al. reported the effectiveness of avelumab maintenance therapy for advanced UC, especially in PD‐L1 positive patients; this was assessed using the SP263 assay scoring algorithm.^32^ Although it seems unlikely that PD‐L1 as a single biomarker will guide administration decisions, the combination of other biomarkers, such as NLR, or continual assessment of PD‐L1 expression, could be potential prognostic markers.

Besides NLR and PD‐L1, younger age was also associated with longer PFS and OS. However, in the KEYNOTE‐045 study, Bellmunt et al. reported that the advantage of pembrolizumab in advanced UC seemed to be consistent in spite of age.^10^ Similarly, among 608 Japanese patients with chemotherapy‐resistant UC, the therapeutic effect of pembrolizumab did not differ with age.^33^ These studies included more cases than this present study and are credible in this context. Notably, in this present study, age was not independently significant in the Cox‐proportional hazards model analysis.

In this study, squamous differentiation was not associated with prognosis. However, in our previous study, squamous differentiation was associated with shorter OS and PFS in UC patients treated with pembrolizumab.^14^ This discrepancy is probably due to the two different types of squamous differentiation. One is the inflamed, well‐keratinized type that occurs from squamous metaplasia, and the other is less keratinized, making it slightly difficult to recognize as squamous differentiation. These two types are biologically distinct.^34^ Notably, we detected a significant modifying effect for PD‐L1 expression on the relationship between squamous differentiation and patient outcomes (*P*_interaction_ = 0.025). Among patients with negative PD‐L1 expression, squamous differentiation was not associated with PFS (data not shown, Log‐rank *P* = 1.00). In contrast, among patients with positive PD‐L1 expression, squamous differentiation tended to have longer PFS (data not shown, Log‐rank *P* = 0.077). In this context, the combination of histological and immunological assessment is important and further study is needed to confirm the association with squamous differentiation and the effectiveness of pembrolizumab.

There are some limitations to this study. First, we set the unique cutoff values of age and NLR to maximize statistical power. In NLR, previous studies set higher cutoff values (3.35 and 4),^28^, ^29^ though these are all consistent with our result. These high cutoff values are not realistic in this present study, as only a few patients had such high NLR. Future studies are needed to determine the optimal cutoff value for age and NLR. A second limitation is the study's retrospective design and the small number of enrolled patients. A larger prospective study is needed to address these limitations.

In conclusion, younger age, lower NLR, and positive PD‐L1 expression were associated with better OS and PFS in UC patients treated with pembrolizumab. Moreover, lower NLR and positive PD‐L1 expression were independently associated with longer OS in multivariate analysis. These results suggest that both host and tumor status can reflect the effectiveness of pembrolizumab among patients with UC.

## ETHICS STATEMENT

This study was approved by the Institutional Review Board of the Saitama Medical University International Medical Center (approval numbers: 20‐129 and 20‐149), and informed consent was acquired from all the patients.

## CONFLICT OF INTEREST

None to declare.

## AUTHOR CONTRIBUTIONS

Yu Miyama designed this study, analyzed the data, made the draft, and revised the manuscript. Go Kaneko designed this study, acquired clinical information, made the draft, and revised the manuscript. Koshiro Nishimoto designed this study, acquired clinical information, analyzed statistical data, and interpreted the data. Masanori Yasuda provided suggestions and revised the manuscript. All authors read and approved the final version of the manuscript.

## Supporting information


Figure S1

Table S1

Data S1

## Data Availability

Data sharing is not applicable to this article as no new data were created or analyzed in this study.
