# Supplementary figures and images for: Lower neutrophil‐to‐lymphocyte ratio and positive programmed cell death ligand‐1 expression are favorable prognostic markers in patients treated with pembrolizumab for urothelial carcinoma
Source: Cancer Med. 2022 Jun 14;11(22):4236–45. doi: 10.1002/cam4.4779 (PMC9678108; doi:10.1002/cam4.4779)

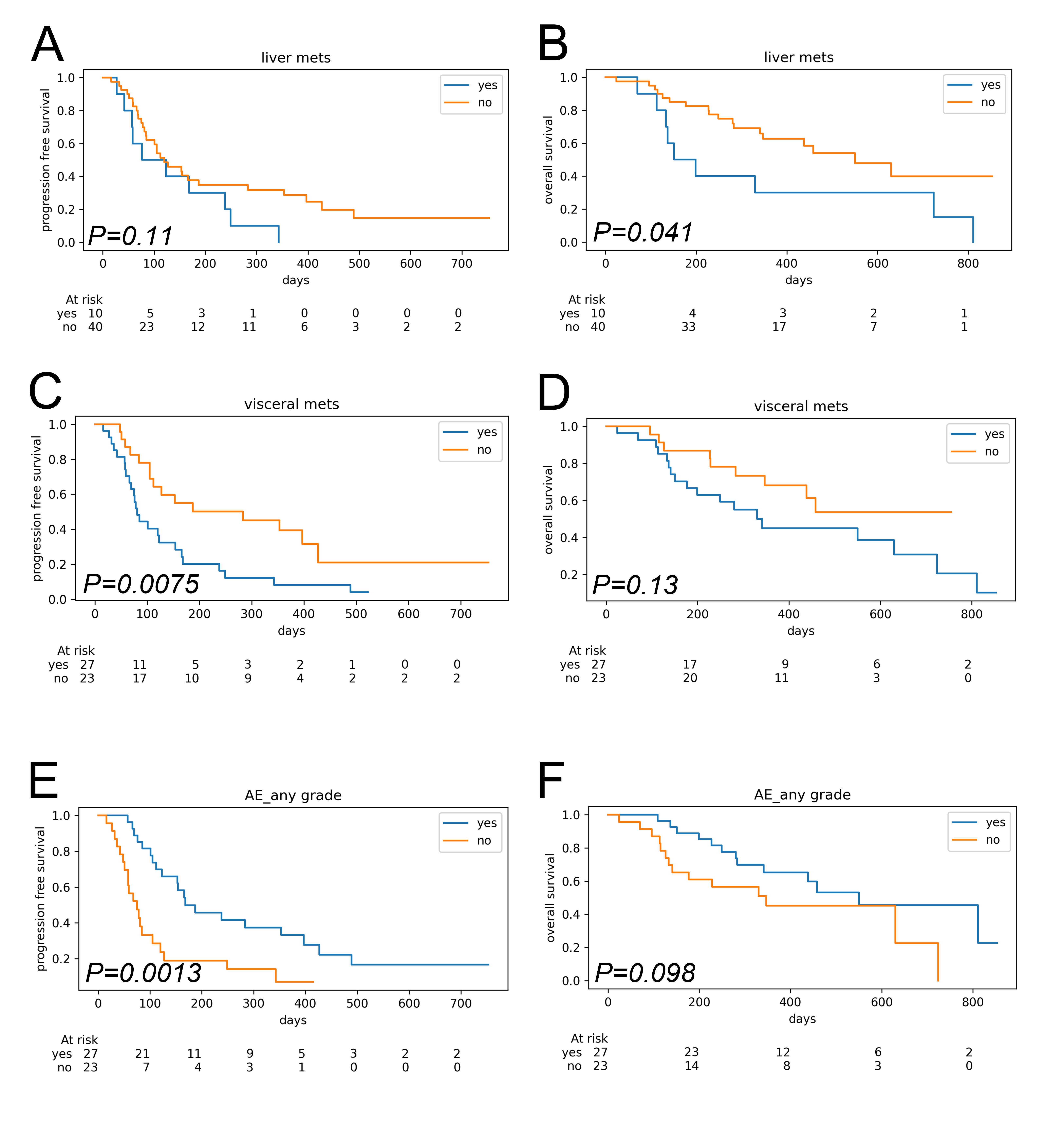

Supplement: Supplementary file 1 — Figure S1 [file CAM4-11-4236-s002.jpg]
