# Supplementary material for: Lower neutrophil‐to‐lymphocyte ratio and positive programmed cell death ligand‐1 expression are favorable prognostic markers in patients treated with pembrolizumab for urothelial carcinoma
Source: Cancer Med. 2022 Jun 14;11(22):4236–45. doi: 10.1002/cam4.4779 (PMC9678108; doi:10.1002/cam4.4779)
Supplement: Supplementary file 3 — Data S1 [file CAM4-11-4236-s003.docx]

Supplementary Figure 1

A. Kaplan-Meier curves for progressive free survival in two groups with or without liver metastasis. Abbreviations: mets, metastasis

B. Kaplan-Meier curves overall survival in two groups with or without liver metastasis.

C. Kaplan-Meier curves for progressive free survival in two groups with or without visceral metastasis.

D. Kaplan-Meier curves for overall survival in two groups with or without adverse events. Abbreviations: AE, adverse events
